# A Lower-Order Oligomer Form of Phage Shock Protein A (PspA) Stably Associates with the Hexameric AAA^+^ Transcription Activator Protein PspF for Negative Regulation

**DOI:** 10.1016/j.jmb.2009.09.055

**Published:** 2009-12-11

**Authors:** Nicolas Joly, Patricia C. Burrows, Christoph Engl, Goran Jovanovic, Martin Buck

**Affiliations:** Division of Biology, Sir Alexander Fleming Building, Imperial College London, Exhibition Road, London SW7 2AZ, UK

**Keywords:** Psp, phage shock protein, bEBP, bacterial enhancer binding protein, PspA, regulatory complex, σ^54^ transcription, PspF, phage shock protein (Psp)

## Abstract

To survive and colonise their various environments, including those used during infection, bacteria have developed a variety of adaptive systems. Amongst these is phage shock protein (Psp) response, which can be induced in *Escherichia coli* upon filamentous phage infection (specifically phage secretin pIV) and by other membrane-damaging agents. The *E. coli* Psp system comprises seven proteins, of which PspA is the central component. PspA is a bifunctional protein that is directly involved in (i) the negative regulation of the *psp*-specific transcriptional activator PspF and (ii) the maintenance of membrane integrity in a mechanism proposed to involve the formation of a 36-mer ring complex. Here we established that the PspA negative regulation of PspF ATPase activity is the result of a cooperative inhibition. We present biochemical evidence showing that an inhibitory PspA–PspF regulatory complex, which has significantly reduced PspF ATPase activity, is composed of around six PspF subunits and six PspA subunits, suggesting that PspA exists in at least two different oligomeric assemblies. We now establish that all four putative helical domains of PspA are critical for the formation of the 36-mer. In contrast, not all four helical domains are required for the formation of the inhibitory PspA–PspF complex. Since a range of initial PspF oligomeric states permit formation of the apparent PspA–PspF dodecameric assembly, we conclude that PspA and PspF demonstrate a strong propensity to self-assemble into a single defined heteromeric regulatory complex.

## Introduction

Bacteria can experience a multitude of stresses, many of which have the potential to affect cell integrity. To survive and colonise their environments, bacteria use a number of adaptive systems. One such system found in a variety of bacterial species is phage shock protein (Psp) response, which can be induced in *Escherichia coli* upon filamentous phage infection (specifically secretin pIV) and by other membrane-damaging agents.^1–3^ The *psp* regulon is conserved amongst enterobacteria and usually comprises the *pspABCDE* operon and the *pspG* gene, transcription of which is activated by PspF (reviewed by Darwin^3^). Under normal growth conditions, PspF activity is negatively regulated by PspA.^4–6^ Following loss of membrane integrity (potentially resulting in dissipation of proton motive force), the negative regulation of PspF activity is lost, enabling PspF to activate σ^54^-dependent transcription from the two *psp* promoters *pspAp* and *pspGp*.^2,7–9^ Maintenance of cell membrane integrity is crucial for bacterial survival, especially when bacteria are exposed to challenging conditions, as is the case with eukaryotic tissue colonisation during infection. For pathogens such as *Salmonella* and *Yersinia*, the Psp response is clearly important for their success as infectious agents.^10,11^ In spite of the significance of the Psp response, the mechanistic basis of its regulation is still poorly understood.

The transcriptional activator PspF is a well-studied member of bacterial enhancer binding proteins (bEBPs) belonging to the AAA^+^ (*A*TPases *a*ssociated with various cellular *a*ctivities) protein family. PspF functions as a higher-order oligomer (a hexamer) and hydrolyses ATP during its role as a transcriptional activator.^12,13^ Although bEBPs are usually composed of three domains (a regulatory domain, an AAA^+^ ATPase domain, and a DNA binding domain), PspF lacks a regulatory domain. Instead, its activity is negatively controlled by PspA acting in trans.^4–7^ As is the case for many bEBPs, the catalytic AAA^+^ domain of PspF (PspF_1–275_) is necessary and sufficient to activate σ^54^-dependent transcription; for PspF_1–275_, its activity has been shown to be regulated by PspA both *in vivo* and *in vitro*.^2,4–6,14,15^

**Fig. 1:**
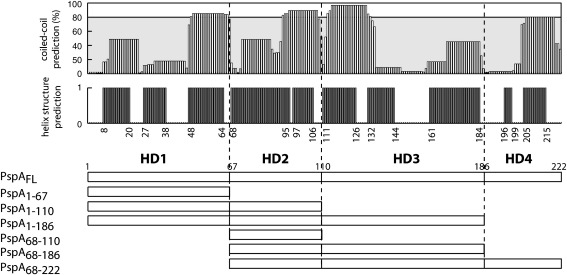
Predicted organisation of PspA_FL_ and the PspA_fragments_ used in this study. PspA is predicted to be a coiled-coil protein consisting of four α-helical domains: HD1 (residues 1–67), HD2 (residues 68–110), HD3 (residues 111–186), and HD4 (residues 187–222), as determined by computer analyses (see Materials and Methods). PspA_FL_ (full length) and PspA_fragments_: PspA_1–67_ (HD1), PspA_68–110_ (HD2), PspA_1–110_ (HD1–HD2), PspA_1–186_ (HD1–HD2–HD3), PspA_68–186_ (HD2–HD3), and PspA_68–222_ (HD2–HD3–HD4), with correspondingly numbered residues, are presented.

PspA is a bifunctional protein that is directly involved in (i) the negative regulation of PspF and (ii) the maintenance of membrane integrity. PspA is predicted to be a coiled-coil protein comprising four α-helical domains (HD1–HD4; Fig. 1)^4,5^ and interacts with the inner membrane proteins PspB and PspC, as well as with PspF (Adams *et al.*^16^ and reviewed by Darwin^3^). The mechanism by which inducing signals (potentially a change in proton motive force or membrane organisation) are sensed and transmitted to PspA is not clear, but appears to usually involve PspBC.^2^ Neither the precise role of each of the Psp proteins nor the mechanistic basis of Psp-mediated membrane repair and PspF negative regulation is known. Apparently, PspB and PspC function as inner membrane sensors of the stress signal, which, during stress conditions, transduce an inducing signal to PspA *via* protein–protein interactions.^3^ “Activation” of PspA somehow alters the PspA–PspF interaction, relieving PspF inhibition and thereby allowing activation of *psp* transcription.

Structural studies have previously established that purified PspA can form a 36-mer ring comprising nine tetramers.^17^ Recently, Standar *et al.* have reported a second PspA oligomeric organisation, called the “PspA scaffold,” that has been proposed to be important for the maintenance of membrane integrity.^18^ Earlier functional studies have determined that PspA acts as an effector by interacting directly with membrane lipids to repair membrane proton leakage.^19^ Elderkin *et al.* demonstrated that PspA acts as a negative regulator by directly binding PspF in an interaction that is dependent on the surface-exposed residue W56 (of PspF).^5,6^ Not all four putative α-helical domains of PspA are required to interact with and to negatively regulate PspF.^5^ PspA inhibition of PspF ATPase activity^6^ probably involves the repositioning of residue N64 (of PspF), which is thought to be involved in the sensing and positioning of the γ-phosphate of ATP.^20^ Analysis of the structural features and activity of PspA can also contribute to an understanding of the function of VIPP1, a PspA homologue that is crucial for thylakoid biogenesis and essential for photosynthesis.^21^ In addition, several common extracytoplasmic stresses known to induce the Psp response in *E. coli* are reported to upregulate PspA expression in other Gram-negative and Gram-positive bacteria, archebacteria, and plants (Bidle *et al.*^22^ and Vrancken *et al.*^23^ and reviewed by Darwin^3^).

The precise relationship between PspA and PspF is poorly understood, especially at the level of the self-association state of PspA and PspF within the inhibitory complexes. Indeed, the organisation of the repressive complex that forms between both proteins is not characterised. Whether the PspA–PspF interaction is dependent on particular nucleotide-bound states of PspF (which might therefore impact on the ATPase activity of PspF) and whether the negative regulation of PspF activity affects the interaction between PspF and σ^54^ remain unknown. To investigate the nature of the molecular mechanism responsible for PspA–PspF negative regulation, we chose an *in vitro* approach using purified proteins. We established that PspA negative regulation of PspF ATPase activity is the result of a cooperative inhibition, and that the PspA/PspF ratio found within the inhibitory PspA–PspF co-complex is near unity. In addition, we have determined what features of PspA and PspF are required for the formation of the PspA–PspF regulatory complex and its inhibited state, and we have demonstrated that some activities of PspF (ultimately used for transcription activation) are not inhibited by PspA.

## Results

### The inhibitory activities of PspA_fragments_ are due to direct binding interactions with PspF

**Fig. 2:**
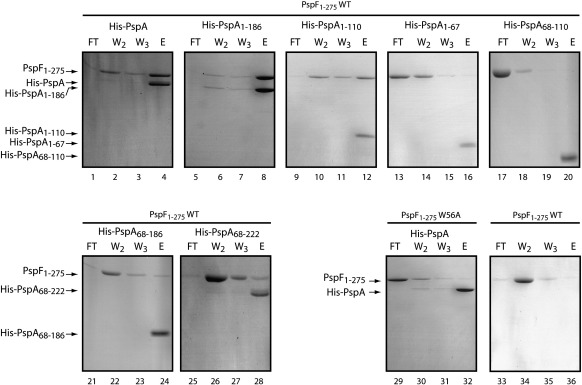
PspA_fragments_ interaction assays. PspA–PspF affinity chromatography using a saturating concentration of PspF_1–275_ applied to a Ni-NTA column preloaded with either His-tagged PspA or PspA_fragments_ (as indicated). The eluted fractions were loaded on SDS-PAGE and stained with Coomassie blue. The amount of PspF_1–275_ after elution in the absence of His-PspA is shown as control. FT: flow through; W2 and W3: wash volumes 2 and 3; E: elution.

The apparent organisation of PspA (predicted to be four α-helical domains, labelled HD1–HD4; see Fig. 1) lends itself to a protein fragmentation approach, with the potential to separate out determinants required for regulatory (modulation of PspF activity) and effector (maintenance of cell membrane integrity) functions by establishing the contributions of each of the HD to the functionalities of PspA. Following purification of PspA_FL_ (full length) and PspA_fragments_ [PspA_1–67_ (HD1), PspA_68–110_ (HD2), PspA_1–110_ (HD1–HD2), PspA_1–186_ (HD1–HD2–HD3), PspA_68–186_ (HD2–HD3), and PspA_68–222_ (HD2–HD3–HD4)] (Fig. 1), we tested the ability of these proteins to interact with the AAA^+^ domain of PspF (herein termed PspF_1–275_WT) and to inhibit its ATPase activity. Previous studies have shown that PspA_FL_, PspA_1–110_, PspA_1–186_, PspA_68–186_, and PspA_68–222_ interact with PspF_1–275_WT^5^ using a native-gel-based nonequilibrium method that is sensitive to buffer and gel-running conditions. One aim of this study was to estimate the stoichiometry of PspA and PspF within the PspA–PspF regulatory complex. To address this issue, we employed an equilibrium method based on affinity chromatography to measure direct binding interactions between PspF and either His-PspA_FL_ or His-PspA_fragments_ (Materials and Methods; Fig. 2). Using this method, we observed an interaction between PspF_1–275_WT and PspA_FL_, PspA_1–110_, PspA_1–186_, PspA_68–186_, and PspA_68–222_, but not with the PspA_1–67_ or PspA_68–110_ fragments. However, we note that the interaction between PspA_68–186_ and PspF_1–275_WT is weaker than the interaction with other PspA_FL_ or PspA_fragments_. Control reactions verified that PspF_1–275_WT could not bind specifically to the column in the absence of His-tagged PspA_FL_ (or PspA_fragments_), and that no clear interactions with either His-PspA_FL_ or His-PspA_fragments_ were detected in the presence of PspF_1–275_W56A (the form of PspF that is unable to interact with PspA).

### PspF ATPase inhibition

**Fig. 3:**
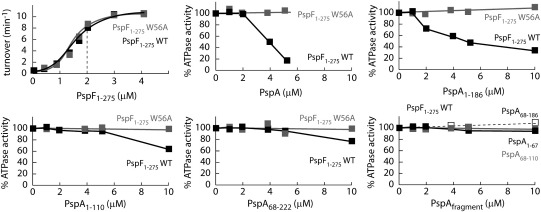
PspA_fragments_ inhibitory effect on PspF ATPase activity. The effect of PspA_fragments_ on the PspF_1–275_ ATPase activity performed at 37 °C in the presence of 2 μM PspF_1–275_ (WT or W56A) and different concentrations of PspA (full length or fragments, as indicated). The results are expressed as percentage of PspF_1–275_WT activity in the absence of PspA.

Having demonstrated that the majority of PspA fragments were able to interact directly with PspF_1–275_WT (but not with PspF_1–275_W56A), we examined whether these interactions were effective in inhibiting PspF ATPase activity. We first determined which concentration of PspF_1–275_WT or W56A to use by establishing for the two proteins the titration curve of the ATPase activity as a function of protein concentration (Fig. 3). We chose to use 2 μM PspF_1–275_WT or W56A for inhibition studies, based on knowledge of the concentration dependency of the ATPase activity.^12^ We then used different concentrations of PspA_FL_ and PspA_fragments_ and observed their effects on the PspF ATPase activity. As shown in Fig. 3, PspA_FL_, PspA_1–186_, PspA_1–110_, and PspA_68–222_ inhibit the PspF ATPase activity with differing efficiencies. Inhibition of the ATPase activity by PspA_1–110_ and PspA_68–222_ is only clearly observed when these fragments are present in large excess over PspF. With the HD4 deletion variant (PspA_1–186_), some ATPase inhibition occurs at a PspF/PspA ratio of 1:1, whereas in the presence of PspA_FL_, significant inhibition is observed at a PspF/PspA ratio of 1:2, suggesting a higher affinity of PspA_1–186_ for PspF than for PspA_FL_ (Fig. 3). However, when the concentration of the PspA_FL_ or PspA_fragments_ is increased (to 1:2 or 1:2.5), the ATPase activity is more strongly inhibited by PspA_FL_ than by PspA_1–186_, suggesting a cooperative inhibition by PspA_FL_, whereas PspA_1–186_ inhibition appears noncooperative. Inhibition of PspF ATPase activity by PspA may therefore require residues 187–222 (HD4) to establish a functional inhibitory cooperativity, but not a cooperativity for binding to PspF.

From these results, we conclude that the different PspA fragments have a range of affinities for PspF and function to decrease (to varying extents) PspF_1–275_WT ATPase activity (Fig. 3). We note that fragments that failed to interact with PspF (PspA_1–67_, PspA_68–110_, and PspA_68–186_) in affinity chromatography experiments (see Fig. 2) were unable to significantly decrease the PspF ATPase activity. Notably, the fragment (PspA_1–186_) that exhibited the strongest affinity for PspF is not the most effective at inhibiting the ATPase activity of PspF, suggesting a mechanism of PspF inhibition more complex than that resulting from simple binding. Indeed, using PspA_FL_, we detect a clear cooperative inhibition of PspF ATPase activity. These results strongly infer that rather than a single HD within PspA being responsible for the inhibitory activity of PspA, PspA HDs function together to efficiently regulate PspF ATPase activity.

### Formation of the regulatory PspA–PspF complex is not dependent on the nucleotide-bound state of PspF

We have previously shown that PspF activity is dependent on the nucleotide-bound state.^12^ Having validated the affinity-based method for assaying the direct binding of PspA to PspF_1–275_WT, we examined whether the nucleotide-bound state of PspF could affect its capacity to bind PspA. Using the affinity binding method, we assayed the interaction between PspF_1–275_WT and either His-PspA_FL_ or His-PspA_fragments_ when either ATP, ATPγS, AMPPNP, ADP, AMP, or no nucleotide was present in the binding and wash buffers. In each case, we observed a very similar (to Fig. 2) PspA–PspF binding interaction profile (data not shown). We therefore conclude that the nucleotide-bound state of PspF does not alter the binding interactions between PspA and PspF required for co-complex formation.

We next investigated directly whether the interaction between PspA and PspF could change the nucleotide binding affinity of PspF. Using a filter-based nucleotide binding assay, we determined the ATP and ADP binding properties of PspF_1–275_WT (as performed by Joly *et al.*^20^^,^^24^) and did not observe any significant difference in the presence of PspA_fragments_ (data not shown). We conclude that PspA binding (to PspF) does not significantly affect the nucleotide binding activity of PspF.

### 36-mer formation is an intrinsic property of PspA_FL_: Determinants of PspA oligomerisation

**Fig. 4:**
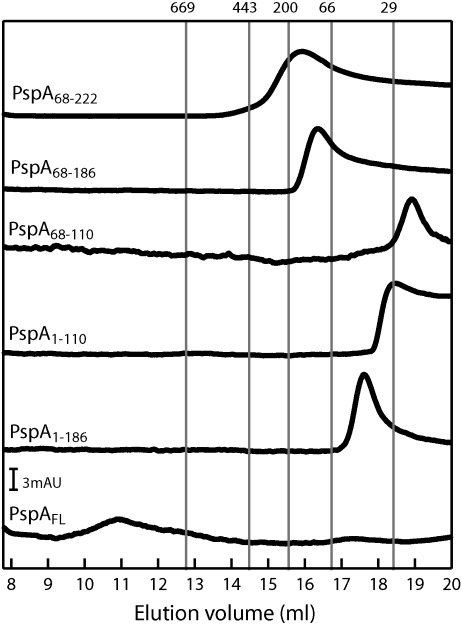
Oligomerisation state of PspA_fragments_. Gel-filtration profiles of 50-μl samples containing 30 μM PspA_fragments_ chromatographed through a Superose 6 column at 4 °C. The column was calibrated with globular proteins (the elution volumes of which are indicated by gray lines): thyroglobulin (669 kDa), apoferritin (443 kDa), β-amylase (200 kDa), bovine serum albumin (66 kDa), and carbonic anhydrase (29 kDa), under the same conditions.

Having established that the majority of purified PspA_fragments_ are functional *in vitro*, we addressed the question of the contribution of each of the putative HDs to the formation of the reported (in the case of PspA_FL_) PspA 36-mer.^17^ Using gel filtration, which separates proteins and protein complexes as a function of their apparent molecular mass based on their apparent stokes radius, we analysed the elution profiles obtained for each of the purified PspA_fragments_ (Fig. 4). Based on the predicted molecular masses of PspA_FL_ (28 kDa), PspA_1–67_ (10 kDa), PspA_68–110_ (8 kDa), PspA_1–110_ (15 kDa), PspA_1–186_ (24 kDa), PspA_68–186_ (17 kDa), and PspA_68–222_ (20 kDa), and the column calibration with globular proteins (see Materials and Methods), we estimated the apparent oligomeric forms of the different PspA_fragments_. For PspA_FL_, we observed a peak with an elution volume corresponding to an apparent 90-mer (Fig. 4). Although this does not appear to directly correlate with the 36-mer in gel filtration, we use globular proteins to calibrate the system; the PspA 36-mer ring is not a globular protein, so based on its apparent stokes radius, we expect the apparent molecular mass of PspA to be much larger than that of a 36-mer. For simplicity, we now refer to the PspA_FL_ oligomeric state as a 36-mer. In the presence of PspA_fragments_ (Fig. 4), we observed elution volumes corresponding to apparent molecular masses lower than that of PspA_FL_: PspA_68–110_ (an apparent dimer), PspA_1–110_ (an apparent dimer), PspA_1–186_ (an apparent dimer), PspA_68–186_ (an apparent hexamer), and PspA_68–222_ (an apparent hexamer).

From the gel-filtration data, we conclude that formation of the apparent higher oligomeric state is an intrinsic property of PspA_FL_ that involves more than one HD. Our data suggest the following: HD2–HD3 are responsible for hexamer formation (PspA_68–186_), and HD1 seems to modulate HD2–HD3-dependent hexamer formation, thus leading to dimers when present (PspA_68–186_ *versus* PspA_1–186_). When HD4 is present with HD1–HD2–HD3 (PspA_1–186_ *versus* PspA_FL_), formation of the 36-mer can occur. We thus conclude that HD4 seems to be required to overcome the negative effect of HD1 on HD2–HD3.

### PspA–PspF regulatory complex has a near-unity protein ratio

**Fig. 5:**
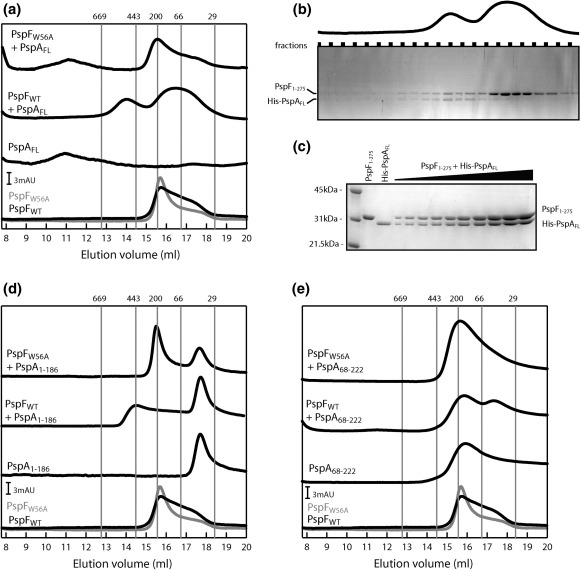
PspA–PspF regulatory complex composition. (a) Gel-filtration profiles of 50-μl samples containing 30 μM PspA_FL_ ± 20 μM PspF_1–275_WT or W56A (as indicated) chromatographed through a Superose 6 column at 4 °C. (b) Fractions corresponding to the elution profile PspF_1–275_WT + PspA_FL_ were loaded on SDS-PAGE and stained by Coomassie blue. (c) SDS-PAGE showing the loading of different quantities of PspA_FL_ and PspF_1–275_WT, Coomassie stained and used as reference for protein ratio estimation. (d) Gel-filtration profiles of 50-μl samples containing 30 μM PspA_1–186_ ± 20 μM PspF_1–275_WT or W56A (as indicated) chromatographed through a Superose 6 column at 4 °C. (e) Gel-filtration profiles of 50-μl samples containing 30 μM PspA_68–222_ ± 20 μM PspF_1–275_WT or W56A (as indicated) chromatographed through a Superose 6 column at 4 °C. The column was calibrated with globular proteins (the elution volumes of which are indicated by gray lines): thyroglobulin (669 kDa), apoferritin (443 kDa), β-amylase (200 kDa), bovine serum albumin (66 kDa), and carbonic anhydrase (29 kDa), under the same conditions.

Having shown that HD1–HD4 of PspA are important for the formation of the native 36-mer (observed with PspA_FL_), and that the majority of PspA_fragments_ were able to interact with PspF and to inhibit its ATPase activity, we next analysed the type of complex formed between PspA and PspF. Clearly, PspA_FL_ can form an apparent higher-order oligomer (36-mer; Fig. 4), but the number of PspA subunits within the PspA–PspF regulatory complex is as yet unknown. To address this question, we performed gel-filtration experiments comparing the different elution profiles of either PspA_FL_ alone or PspF_1–275_WT alone to that of a mixture of PspA_FL_ and PspF_1–275_WT (incubated for 15 min at 4 °C prior to loading on the gel-filtration column; Fig. 5a). In the mixed sample, we observed a distinct PspF_1–275_WT·PspA_FL_ complex (apparent molecular mass, 384 kDa), which elutes with an apparent molecular mass lower than that of PspA_FL_ alone (36-mer) yet higher than that of PspF alone (hexamer; Fig. 5a). Analysis of the fractions by SDS-PAGE demonstrated the presence of PspA_FL_ and PspF_1–275_WT proteins in this new peak (Fig. 5b). To estimate the ratio of PspA–PspF protein present in the complex, we used SDS-PAGE and loaded different concentrations of His-PspA_FL_ and PspF_1–275_WT proteins (Fig. 5c; data not shown). Although the quantities of the proteins loaded in the gel were very similar, the intensities of Coomassie staining appear to be different (however, we note that PspF_1–275_WT is more preferentially stained than His-PspA_FL_). A similar difference in stain intensity is seen in the fraction corresponding to the peak containing the PspA–PspF complex, suggesting a 1:1 PspF/PspA protein ratio in this complex. Based on the estimated protein ratios from the stained SDS-PAGE and the apparent molecular mass of the complex, we estimated the protein stoichiometry of this new complex to be close to six PspA_FL_ subunits and six PspF_1–275_WT subunits (Fig. 5b). No PspF was detected in the void volume fraction, suggesting that the PspF is only bound to PspA in a co-complex that is close to a dodecamer. As negative control, we used PspF_1–275_W56A (which cannot interact with PspA) in the gel-filtration assays and were unable to observe the formation of the co-complex with PspA, since the gel-filtration profiles remained identical with those of PspF_1–275_W56A and PspA_FL_ alone (Fig. 5a).

### PspA_fragments_ form PspA–PspF regulatory co-complexes

We then tested whether discrete regulatory co-complexes could be observed in the presence of PspA_fragments_ using the same gel-filtration approach as with PspA_1–186_ mixed with PspF_1–275_WT. In this case, we observed a similar regulatory co-complex PspF_1–275_WT·PspA_1–186_ (apparent molecular mass, 358 kDa) comparable to that seen with PspA_FL_ (apparent molecular mass, 384 kDa; Fig. 5a and d). Interestingly, the difference between the apparent molecular masses of the two co-complexes (384–358 kDa = 26 kDa) corresponds to six times the molecular mass of HD4, which is absent in PspA_1–186_ (6 × 4 kDa = 24 kDa). We conclude that PspA_1–186_ forms a regulatory complex with PspF at a molar ratio close to 6:6. Therefore, formation of the regulatory co-complex appears to be independent of the ability of PspA to form the higher-order oligomeric complex (36-mer), since PspA_1–186_ is an apparent dimer, suggesting some form of codependence between PspA and PspF for the formation and organisation of the hetero near-dodecameric co-complex. Similar results were obtained with the PspA_1–110_ fragment, and the co-complex formed with PspF was again shifted (in terms of elution volume)—this time the difference in the apparent molecular mass corresponded to six times the molecular masses of HD3–HD4 (which are absent in the PspA_1–110_ fragment; data not shown).

### HD1 is important for the formation of the native PspA–PspF regulatory co-complex

We have shown that, with the exception of HD1 alone (PspA_1–67_) or HD2 alone (PspA_68–110_), all the PspA_fragments_ tested were able to interact with PspF and to inhibit its ATPase activity. However, we note that HD2–HD3 (PspA_68–186_) alone only weakly interacts with PspF and has no inhibitory effect on ATPase activity. Surprisingly, when we conducted the gel-filtration experiments using PspA_68–222_ (Fig. 5e) or PspA_68–186_ (data not shown), we observed a PspF_1–275_WT·PspA_68–222_ co-complex very different from that observed with PspA_FL_. The apparent molecular mass of the PspF_1–275_WT·PspA_68–222_ co-complex is 55 kDa, consistent with a heterodimer (1:1) and not with a heterododecamer (6:6). From these observations, we propose that HD1 of PspA is not necessary for binding PspF, but plays an important role in the formation of the regulatory hetero near-dodecameric co-complex observed between PspF and PspA_FL_.

### PspA–PspF regulatory co-complex is not dependent on the initial oligomeric state of PspF

**Fig. 6:**
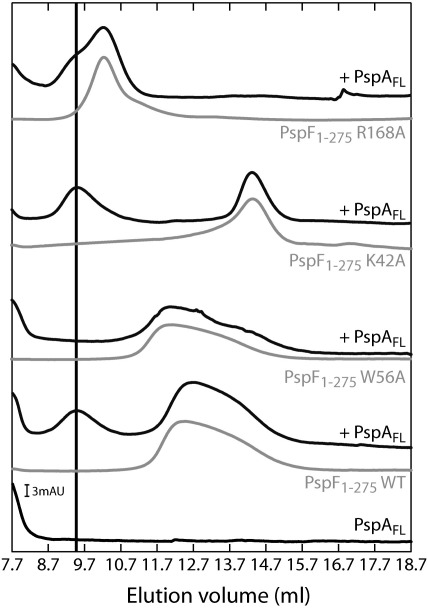
PspA–PspF regulatory complex formation is not dependent on the oligomeric state of PspF. Gel-filtration profiles of 50-μl samples containing 30 μM PspA_FL_ ± 20 μM PspF_1–275_ (WT, W56A, K42A, or R168A) were chromatographed through a Superdex 200 column at 4 °C. The position of the PspA–PspF co-complex is indicated by the black line. In this column, PspA alone elutes in the void volume. The column was calibrated with globular proteins: thyroglobulin (669 kDa), apoferritin (443 kDa), β-amylase (200 kDa), bovine serum albumin (66 kDa), and carbonic anhydrase (29 kDa), under the same conditions.

PspA_FL_, PspA_1–186_, and PspA_1–110_ bind PspF_1–275_WT to form regulatory hetero near-dodecameric co-complexes independent of the ability of PspA_1–186_ or PspA_1–110_ to form higher-order oligomeric structures. The organisation of PspA in the regulatory co-complex with PspF clearly cannot be that of the 36-mer form. We next addressed whether the oligomeric state of PspF could affect the formation of the regulatory co-complex. Using a Superdex 200 column to achieve a greater resolution between the PspA–PspF regulatory co-complex and the PspF hexamer elution volumes (compare Figs. 5a and 6), we initially reproduced the observation that the PspA–PspF co-complex consists of an apparent 6:6 ratio of PspA/PspF (Fig. 6). We then performed similar experiments with different forms of PspF previously reported as monomeric/dimeric (PspF_1–275_K42A) or constitutively hexameric (PspF_1–275_R168A) species.^25^ Notably, the PspF_1–275_K42A and PspF_1–275_R168A co-complexes formed with PspA eluted at a volume similar to that observed with PspF_1–275_WT. We conclude that the initial oligomeric state of PspF does not prevent the formation of the near-dodecameric PspA–PspF regulatory co-complex.

### PspA does not inhibit PspF from interacting with σ^54^

**Fig. 7:**
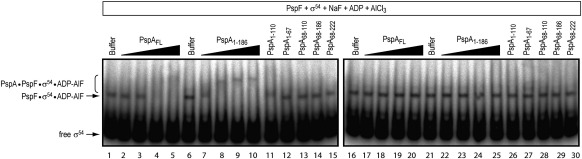
PspA–PspF regulatory complex can bind σ^54^. Native gel migration of “trapped” radiolabelled σ^54^ complexes containing PspF_1–275_WT or PspF_1–275_W56A and PspA (full length or fragments, as indicated). DNA complexes were detected by autoradiography.

PspF transiently associates with the closed promoter complex in its ATP-bound state, but can be stably “trapped” in association with the closed complex using the ATP transition state analogue ADP-AlF.^26^ We considered whether PspA negative regulation might function by preventing PspF from interacting with σ^54^. To address this possibility, we performed “trapping” experiments using radiolabelled σ^54^ to follow the formation of the PspF·σ^54^·ADP-AlF complex (Fig. 7). We observed that, in the presence of PspA_FL_ or PspA_1–186_, the PspF·σ^54^·ADP-AlF complex was supershifted in a PspA_FL_ or PspA_1–186_ concentration-dependent manner, in direct agreement with the different affinities of these fragments for PspF determined in the ATPase inhibition assay. The PspA-dependent supershifts were dependent on residue W56 and were therefore the result of specific PspA–PspF interactions. We conclude that PspA does not obviously inhibit the binding interaction between PspF and σ^54^, suggesting that PspA, when bound to PspF, does not occlude the σ^54^ binding site to achieve inhibition.

## Discussion

Studying the contributions of the PspA HDs to (i) self-association, (ii) PspF co-complex formation, and (iii) PspF negative regulation is important in establishing the switching mechanism between the regulatory function and the effector function of PspA. Previous studies have shown that PspA can form different oligomeric complexes *in vitro* (a 36-mer and a “scaffold complex”^7,18^), and that the PspA 36-mer can directly interact with membrane lipids to restore membrane integrity and to prevent proton leakage,^19^ unlike the monomeric form of PspA. In this study, using an *in vitro* approach, we demonstrate that PspA negative regulation of PspF ATPase activity is the result of a cooperative inhibition. We also establish that all four putative HDs of PspA are important in supporting the formation of the higher oligomeric form of PspA (36-mer). We show that the regulatory complex formed between PspA and PspF is near-dodecameric and likely comprises six PspA subunits and six PspF subunits. In addition, we establish that formation of this co-complex is not dependent on the initial oligomeric state of PspF (and/or the nucleotide-bound state of PspF) or on the ability of PspA to form the 36-mer, but depends largely on the presence of HD4 (of PspA). Taken together, these results suggest that regulation of PspA activity could be achieved by altering the level of PspA self-association, which in turn is dependent on particular HDs. Interactions between different PspF and PspA oligomeric states (both higher and lower) yielded a near-dodecameric co-complex, suggesting that the final heteromeric complex arrived at is relatively stable and is the favoured configuration.

### Regulation of the PspA–PspF interaction

**Fig. 8:**
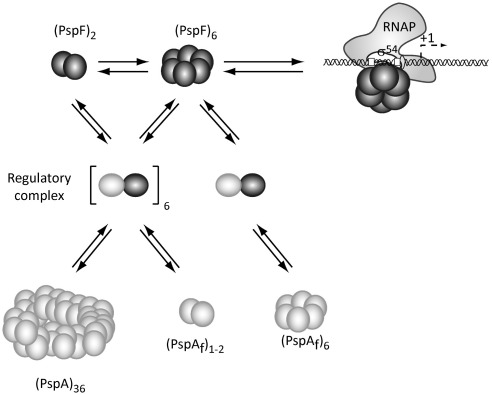
Summary of PspA and PspA–PspF interactions. PspF exists in equilibrium between a dimer and a hexamer. The PspF hexamer is active for ATPase and σ^54^-dependent transcription activation of the *psp* regulon. PspA can form a higher-order oligomer (36-mer) but, in the presence of PspF, forms a much smaller co-complex. The PspA–PspF regulatory co-complex is composed of approximately six PspA subunits and six PspF subunits, and formation of this complex is not dependent on the ability of PspA to form a 36-mer, but is dependent on PspA HD1.

Using the fragmentation approach, we have shown that all four putative HDs of PspA are important in the formation of the 36-mer (thought to represent the effector complex). We show that deleting at least one of these HDs drastically affects the organisation of the PspA oligomer (i.e., the 36-mer is no longer apparent). From the gel-filtration results, we propose that HD1 acts as a negative regulator of the oligomerisation activity of HD2–HD3 (of PspA), which form a hexamer in the absence of HD1 and a dimer in its presence. In addition, HD4 has a direct effect on the oligomerisation of PspA when HD1 is present, suggesting that the inhibitory activity of HD1 is suppressed by HD4. We have shown that the binding interactions between PspA and PspF required for co-complex formation are not dependent on the ability of PspA to form the 36-mer. Furthermore, the resulting co-complexes had reduced PspF ATPase activity, suggesting that formation of the PspA 36-mer is not necessary for the formation of the repressive co-complex between PspA and PspF (Fig. 8). The PspA 36-mer is most probably the effector complex and is not expected to have a direct regulatory role with respect to control of PspF ATPase activity.

### Interconnectivity between PspA–PspF in the regulatory co-complex organisation

We demonstrate that PspA interacts with PspF, and that this interaction promotes the formation of a hetero near-dodecamer co-complex likely formed of six PspA subunits and six PspF subunits. Using PspF variants differing in their degree of self-association, we show that the initial oligomeric state of PspF does not prevent the formation of the final regulatory co-complex. This last point is interesting and significant, since we have previously established that PspF is in equilibrium between an inactive dimeric form and an active hexameric form.^12,25^ Hence, it was formally possible that regulation of PspF ATPase activity by PspA could have occurred at the level of regulation of the self-association activity of PspF (since the hexameric form is required for activity), as is the case for other EBPs containing an amino-terminal regulatory domain.^27^ Strikingly, since we now show that the regulatory complex is formed of approximately six PspF subunits and six PspA subunits, it strongly argues against an ATPase inhibition mechanism in which PspA exerts negative control by forcing PspF to remain dimeric. The organisation of the PspF subunits in the PspA–PspF co-complex is probably sufficiently different from the one in the active PspF hexamer to cause loss of ATPase activity, but not the ability to interact with σ^54^ (where PspF_1–275_ is expected to be a hexameric ring^28^). We show that PspF can affect the organisation of PspA in the regulatory co-complex, and that HD1 of PspA also affects the organisation of the PspA–PspF complex. Interestingly, the PspA_68–222_ fragment (which is missing HD1) is able to interact with PspF and to inhibit its ATPase activity, but the stable PspA–PspF complex formed is an apparent heterodimer. These data suggest that PspA can indeed change the PspF–PspF oligomerisation interface (used for forming the ATPase active PspF hexamer) and, in so doing, alters the presentation of key determinants required for ATPase activity that are also localised at this interface.^13,29^ These results suggest a complex codependency between PspA and PspF that leads to a stable near-dodecameric co-complex with inhibited PspF ATPase activity. The interconnectivity between PspA and PspF in the formation of a regulatory dodecameric co-complex with six PspF subunits may be an important requirement for the rapid release of PspA-imposed negative control.

### The role of PspA in Psp regulation and effector function

Our new observations provide insights into the control of the Psp system, which uses a regulation mechanism known to be important in bacterial virulence.^3,10,11,30^ Under nonstress growth conditions, PspA negatively regulates PspF activity to maintain a low-level expression of the Psp system prior to its induction. Under stress conditions, the *psp*-inducing signal is expected to promote a change in the PspA–PspF interaction. PspF would then be able to activate transcription of the *psp* regulon. The amount of PspA in the cell will increase markedly, whereas the amount of PspF remains constant.^9,31^ Increasing levels of PspA (under inducing conditions) would promote formation of the large 36-mer PspA complex, which is proposed to interact with the inner membrane to prevent proton leakage.^19^ The oligomeric form of PspA under inducing conditions may be such that it can no longer inhibit PspF functionality. A signal to maintain the nonrepressive form of PspA probably exists, and we speculate that additional factors such as PspB and PspC could be involved in preventing the repressive PspA–PspF co-complex from forming or in converting a repressive PspA–PspF complex into a nonrepressive one by causing its reorganisation. PspB and PspC, which directly interact with PspA, have a crucial role in inducing the Psp response as positive control proteins and, like PspA, are upregulated under *psp*-inducing conditions.^3^ It is therefore formally possible that a PspABC complex “decays” to allow the formation of the repressive PspA–PspF regulatory co-complex. The *in vitro* cooperativity inhibition of PspF ATPase activity observed as a function of PspA concentration may be related to setting the rate of transition between the repressed state and the nonrepressed state of PspF.

## Materials and Methods

### Plasmids

pET28b^+^-based plasmids pSLE18A, pSLEPspA1-67, pSLEPspA1-110, pSLEPspA1-186, pSLEPspA68-186, and pSLEPspA68-222 encode the full-length PspA and PspA fragments PspA_1–67_, PspA_1–110_, PspA_1–186_, PspA_68–186_, and PspA_68–222_, respectively, as amino-terminal 6His-fused proteins.^5^ To construct the plasmid pGJ52 encoding PspA_68–110_ fragment, we used the template pPB10, the primers PspA-68-F-NdeI 5′-CATATGGAGGTTGAATGGCAGGAAAAAGCCG-3′ and PspA-110-R-BamHI 5′-GGATCCCAGCGTCACTTCATGTTCCAGGG-3′, and PCR. The DNA fragment obtained contained a NdeI restriction site and a start codon at its 5′-end, and a BamHI and stop codon at its 3′-end flanking the *pspA* sequence encoding PspA_68–110_. This NdeI-BamHI DNA fragment was cloned into a pGEM-T Easy vector and then subcloned into pET28b^+^ digested with NdeI-BamHI, creating pGJ52.

### Nucleotides

ATP, AMPPMP, ATPγS, AMP, and ADP were obtained from Sigma at the highest purity level available. Radiolabelled nucleotides were purchased from Perkin-Elmer.

### Protein structure prediction

We submitted the amino acid sequence of *E. coli* PspA to different structure prediction programmes (nnPredict, Predator, and Coiled-coil prediction), the results of which are summarised in Fig. 1. Note that we only assigned the “predicted structure” for each amino acid when all three software programs gave the same putative structure.

### Protein purification

Full-length PspA or fragments were purified by affinity chromatography, as described by Elderkin *et al.*^5^ The purified protein was dialysed overnight at 4 °C against a final storage buffer [20 mM Tris–HCl (pH 7.5), 200 mM NaCl, 75 mM NaSCN, 0.005% 3-[(3-cholamidopropyl)dimethylammonio]propanesulfonic acid, and 5% glycerol] and frozen at − 80 °C. PspF_1–275_WT and variants W56A, K42A, and R168A encoded by pET28b^+^-based plasmids were purified as described in Joly *et al.*^12^ The proteins were stored in buffer [20 mM Tris–HCl (pH 8.0), 50 mM NaCl, 1 mM DTT, 0.1 mM ethylenediaminetetraacetic acid, and 5% glycerol] and frozen at − 80 °C. σ^54^ and HMK-σ^54^ were purified as described by Cannon *et al.*^32^ and stored at − 80 °C in 20 mM Tris–HCl (pH 8.0), 200 mM NaCl, 0.1 mM ethylenediaminetetraacetic acid, 1 mM DTT, and 50% glycerol. *E. coli* core RNAP enzyme (Epicentre) was purchased from Cambio. Protein concentration was estimated using the Lowry method.^33^

### Affinity chromatography with immobilised His-PspA_FL_ or His-PspA_fragments_

Affinity chromatography was performed at room temperature in Micro Biospin® Bio-Rad columns packed with 50 μl of Ni-NTA agarose (Qiagen). His-PspA_FL_ or His-PspA_fragments_ (500 μl of 6 μM) were bound to the columns. The columns were then washed with 20 vol of buffer A containing 20 mM Tris–HCl (pH 8.0), 50 mM NaCl, and 15 mM MgCl_2_ ± 1 mM nucleotide (AMPPNP, ATPγS, ATP, ADP, or AMP). PspF_1–275_WT or W56A (200 μl at 20 μM) was added, and the unbound protein was removed by washing with 5× 2 vol of buffer A plus 40 mM imidazole ± 1 mM nucleotide. His-tagged PspA (full length or fragments) was eluted with 2× 2 vol of buffer A containing 500 mM imidazole. One-hundred-microliter fractions were collected, and 20 μl was analysed by 12% SDS-PAGE (acrylamide/bisacrylamide, 37.5:1).

### ATPase activity

ATPase activity assays were performed at a final volume of 10 μl in a buffer containing 35 mM Tris-acetate (pH 8.0), 70 mM potassium acetate, 15 mM magnesium acetate, 19 mM ammonium acetate, 0.7 mM DTT, and 1 μM PspF_1–275_ ± PspA full-length or fragments (as indicated). The mix was incubated at 37 °C for 10 min, and the reaction was started by adding 3 μl of an ATP solution containing 0.6 μCi/μl [α-^32^P]ATP (3000 Ci/mmol) and 0.1 mM ATP. The reactions were incubated at 37 °C for different times and then quenched by addition of 5 vol of 2 M formic acid. [α-^32^P]ADP was separated from ATP by thin-layer chromatography, and radiolabelled ADP and ATP were measured by PhosphorImager (Fuji FLA-5000) and analysed using Aida software. Activity is expressed as percentage turnover compared to PspF_1–275_WT. All experiments were carried out independently at least in triplicate, and fluctuations of turnover values were maximally 10%.

### Gel filtration through Superdex 200 or Superose 6

PspF_1–275_WT or variants and PspA_FL_ or PspA_fragments_ (as indicated) were incubated for 10 min at 4 °C in a buffer containing 20 mM Tris–HCl (pH 8.0), 50 mM NaCl, and 15 mM MgCl_2_. Fifty-microliter samples were then injected onto a Superdex 200 column (10 mm × 300 mm, 24 ml; GE Healthcare) or a Superose 6 column (10 mm × 300 mm, 24 ml; GE Healthcare) installed on an AKTA system (GE Healthcare) and equilibrated with the sample buffer. Chromatography was performed at 4 °C at a flow rate of 0.5 ml/min, and columns were calibrated with the globular proteins thyroglobulin (669 kDa), apoferritin (443 kDa), β-amylase (200 kDa), bovine serum albumin (66 kDa), and carbonic anhydrase (29 kDa). All experiments were repeated at least two times, and the elution profiles obtained were similar. Proteins were detected at a wavelength of 280 nm (mAU: milli absorbance unit at 280 nm).

### Native gel mobility shift assay

Gel mobility shift assays were conducted to detect protein–protein or protein–DNA complexes. Assays were performed at a final volume of 10 μl containing 10 mM Tris-acetate (pH 8.0), 50 mM potassium acetate, 8 mM magnesium acetate, 0.1 mM DTT, and 4 mM ADP ± NaF (5 mM) ± HMK-σ^54^ (radiolabelled) (1 μM). Where required, PspF_1–275_WT or W56A (2 μM), with or without different concentrations of full-length PspA or PspA fragments (as indicated), was added and incubated for 5 min at 37 °C. After addition of 0.4 mM AlCl_3_, the reaction mixture was incubated for 20 min at 37 °C to allow *in situ* formation of metal–fluoride analogues. Complexes were analysed on a native 4.5% polyacrylamide (acrylamide/bisacrylamide, 37.5:1) gel run in TG buffer [25 mM Tris–HCl (pH 8.3) and 192 mM glycine]. Radiolabelled protein complexes were detected by PhosphorImager (Fuji Bas-5000) and analysed using the Aida software.
